# An Overview of Different Strategies to Recreate the Physiological Environment in Experimental Erythropoiesis

**DOI:** 10.3390/ijms21155263

**Published:** 2020-07-24

**Authors:** Cécile Deleschaux, Martina Moras, Sophie D. Lefevre, Mariano A. Ostuni

**Affiliations:** 1 INSERM, Biologie Intégrée du Globule Rouge/UMR_S1134/BIGR, Université de Paris, F-75006 Paris, France; cecile.deleschaux@inserm.fr (C.D.); martina.moras@inserm.fr (M.M.); sophie.lefevre@inserm.fr (S.D.L.); 2 INSERM, Biologie Intégrée du Globule Rouge/UMR_S1134/BIGR, Université des Antilles, F-75006 Paris, France; 3 INTS, Département de Recherche sur les Globules Rouges/DRGR, F-75015 Paris, France

**Keywords:** erythropoiesis, bone marrow, physiological environment, mesenchymal stromal cells, erythroblastic island, experimental approaches

## Abstract

Human erythropoiesis is a complex process leading to the production of mature, enucleated erythrocytes (RBCs). It occurs mainly at bone marrow (BM), where hematopoietic stem cells (HSCs) are engaged in the early erythroid differentiation to commit into erythroid progenitor cells (burst-forming unit erythroid (BFU-E) and colony-forming unit erythroid (CFU-E)). Then, during the terminal differentiation, several erythropoietin-induced signaling pathways trigger the differentiation of CFU-E on successive stages from pro-erythroblast to reticulocytes. The latter are released into the circulation, finalizing their maturation into functional RBCs. This process is finely regulated by the physiological environment including the erythroblast-macrophage interaction in the erythroblastic island (EBI). Several human diseases have been associated with ineffective erythropoiesis, either by a defective or an excessive production of RBCs, as well as an increase or a hemoglobinization defect. Fully understanding the production of mature red blood cells is crucial for the comprehension of erythroid pathologies as well as to the field of transfusion. Many experimental approaches have been carried out to achieve a complete differentiation in vitro to produce functional biconcave mature RBCs. However, the various protocols usually fail to achieve enough quantities of completely mature RBCs. In this review, we focus on the evolution of erythropoiesis studies over the years, taking special interest in efforts that were made to include the microenvironment and erythroblastic islands paradigm. These more physiological approaches will contribute to a deeper comprehension of erythropoiesis, improve the treatment of dyserythropoietic disorders, and break through the barriers in massive RBCs production for transfusion.

## 1. Introduction

Erythropoiesis is a complex process involving several stages of differentiation, resulting in the production of mature erythrocytes. Hematopoietic stem cells (HSCs) localized in the bone marrow (BM) are the firsts progenitors engaged in this process, giving rise to the erythroid lineage [1,2]. Two well-characterized differentiation steps are required to produce mature red blood cells (RBCs). During the early erythroid differentiation, HSCs commit into erythroid progenitor called burst-forming unit erythroid (BFU-E) and then into colony-forming unit erythroid (CFU-E). Then, during the terminal erythroid differentiation, erythropoietin (Epo)-induced signaling transduction pathways induce the successive differentiation from pro-erythroblasts (Pro-E) to basophilic (Baso-E), polychromatic (Poly-E), and orthochromatic (Ortho-E) erythroblasts. Specifically in mammals, the Ortho-E expels its nucleus resulting in an enucleated reticulocyte and a pyrenocyte, the nucleus surrounded by a thin layer of cytoplasm. Several evidences pointed out that proliferation, differentiation, and enucleation of erythroblasts are strongly regulated by the interaction between the erythroblasts and a macrophage, forming a specialized microenvironment called the erythroblastic islands [3,4]. Besides morphological changes, terminal phase is also characterized by cellular processes including progressive hemoglobinization, chromatin condensation, nuclear pyknosis, changes in the synthesis and assembly membrane components, and organelles clearance [5,6]. Thereafter, the reticulocytes are released into the circulation and initiate their maturation to become functional RBCs.

Ineffective erythropoiesis links with either an altered production of RBCs or a hemoglobinization defect, leading to empty cells. RBCs production could be altered by lower HSC generation, deficient erythroblasts maturation, and/or increased erythroblasts apoptosis.

Among human hematological pathologies associated with ineffective erythropoiesis, we can mention: (i) myelodysplastic syndromes (MDS), which are characterized by ineffective hematopoiesis and peripheral cytopenia and classified according to the low to high risk to develop myeloid leukemia (Reviewed [7]); and (ii) thalassemia and sickle cell disease (SCD), the two most common erythroid monogenic hemoglobinopathies, which are also characterized by chronic anemia, bone strokes, and organ failure (reviewed in References [8,9,10]). Chronic anemia usually induces exacerbated erythrocyte production leading to splenic sequestration and splenomegaly.

However, involved molecular abnormalities are heterogeneous and affect erythropoiesis hierarchy in different ways. For instance, different studies have shown that in MDS, anomalies are detected at early erythroid differentiation giving a clonal HSC abnormality, but also at terminal differentiation showing accumulation of mitochondria leading to apoptosis [11,12]. Moreover, terminal erythropoiesis is also affected in thalassemia and SCD. Both are caused by a defect in hemoglobin synthesis and result in anemia [8,13]. Sign of its complexity, and despite a lot of studies, the complete understanding of erythroid disorders remains unclear.

Finally, understanding the production of mature red blood cells is also crucial in the field of transfusion. Many experimental approaches have been carried out to achieve a complete differentiation in vitro to produce fully functional biconcave mature RBCs. However, the various protocols usually fail to achieve enough quantities of completely mature red blood cells. In this review, we focus on the evolution of erythropoiesis studies over the years, taking special interest in efforts that were made to include the microenvironment and erythroblastic islands paradigm. These more physiological approaches will contribute to a deeper comprehension of erythropoiesis, improve the treatment of dyserythropoietic disorders, and break through the barriers in massive RBCs production for transfusion.

## 2. Evolution of Experimental Approaches

### 2.1. Hematopoietic Stem Cells

**Figure 1:**
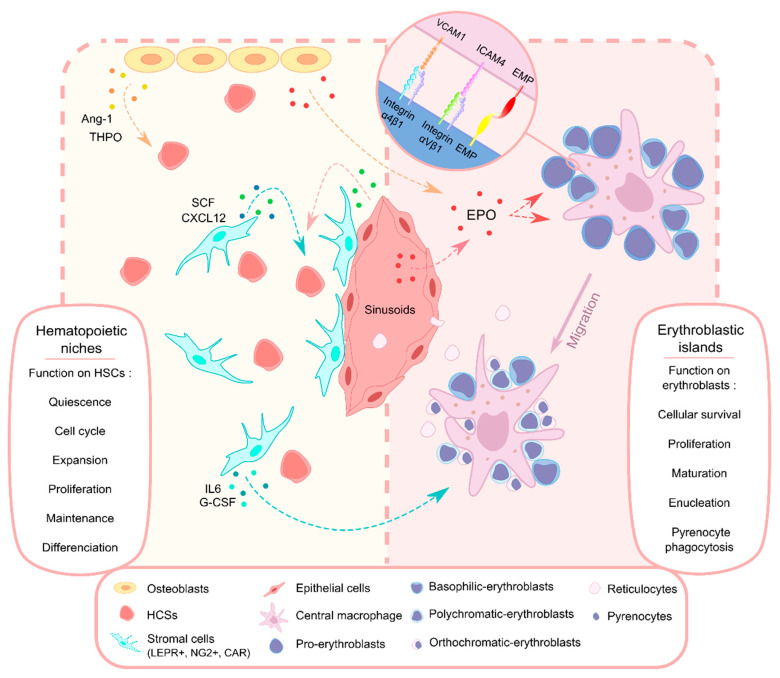
Summary of cell to cell dialog within the erythroblastic niche. Left panel: osteoclasts, stromal cells, and epithelial cells from the sinusoids act simultaneously to maintain the pool of undifferentiated HSC. Right panel: these cells produce cytokines inducing the differentiation of erythroblasts which interact with macrophages within the erythroblastic island, promoting proliferation and terminal differentiation.

To conduct in vitro erythropoiesis, most researchers take advantage of HSC CD34^+^ cells located in peripheral blood (PB), umbilical cord blood (CB), fetal liver, and bone marrow (BM). As reviewed by Sun et al., this method has been used extensively and optimized over the last 50 years [14]. Discovery of growth factors and cytokines governing the proliferation/differentiation balance, such as EPO and stem cell factor (SCF) (Figure 1), has greatly increased the yield of erythrocyte production [15,16]. However, the protocols developed differ according to the composition of the cytokines cocktail, the concentration of the cells, the days of culture, and the number of phases [14]. Mostly, an increase in expansion is gradually observed, but some limitations appear preventing a complete reconstitution of the erythropoiesis in vivo: low rate of enucleation and persistence of fetal hemoglobin (HbF). The proliferation rate remains variable depending on the origin of HSC, even if there are no proteomic differences between cells from cord blood or peripheral blood [17,18,19]. Recently, the development of a Good Manufacturing Practice (GMP)-grade medium allowed the increased production of enucleated erythrocytes containing adult hemoglobin [20]. Based on Cellquin medium, GPM-grade medium includes growth factors, hormone combinations, and human protein-based media [21]. In addition, HSCs are also widely used in co-culture to mimic microenvironment and mouse transplantation, making them an essential resource in hematological research.

### 2.2. Inducible Pluripotent Stem Cell (iPSC)

Embryonic stem cells (ESCs) were used to study erythropoiesis in mammals [22,23]; however, use of human embryonic stem cells (hESCs) raises strong ethical issues. The discovery of the Yamanaka factors (Oct3/4, Sox2, Klf4, c-Myc) and thus the development of somatic cells reprogramming to iPSCs, provides new possibilities to generate erythroid progenitors, achieve a full erythropoiesis, and gives us the opportunity to study erythropoiesis from pathological blood samples [24,25]. Subsequently, many studies have succeeded in generating hematopoietic cells and getting them into the erythroid lineage with iPSCs derived from healthy or pathological patient but, as with HSCs, the enucleation rate remains low and with predominant fetal hemoglobin expression [26,27,28,29]. However, Uchida’s team has recently generated iPSCs from sac-derived erythroid cells, which were able to express adult hemoglobin. They have also generated iPSCs derived from fibroblast, bone marrow stromal cells (BMSCs), or peripheral blood erythroid progenitors (EPs) from healthy or sickle cell disease patients expressing beta-globin [30,31]. The iPSCs, in addition to their renewal capacity, are interesting models for transgenics strains. In 2017, Yang et al. managed to integrate a tamoxifen-inducible KLF1 (Erythroid Kruppel-like factor 1) gene into the safe harbor *AAVS1* locus and successfully created an iPSCs transgenic line with significantly increased percentage of enucleation [32]. KLF1 has been previously described as an important transcription factor during the erythroid differentiation and enucleation as well as GATA1 [33]. Even with updated cell culture, the differentiation protocols of the iPSCs still need to be improved to use this tool in an optimal way.

### 2.3. Mice Models

The use of mouse models to study different biological mechanisms is a well-established approach providing many advantages including the specifically organ-targeted gene invalidation and the complexity of a physiological in vivo context. However, despite it has led to the discovery of several important erythropoiesis mechanisms, there are fundamental differences in the physiological pathways of terminal RBCs maturation as well as at the transcriptomic level during differentiation. [34,35].

Due to the limitation of other in vitro approaches to achieve high levels of mature RBCs, in vivo protocols were conducted to recreate whole erythropoiesis, with immunodeficient “humanized” mice. In 2002, transplantation of human early erythroid precursors into sublethally irradiated NOD-SCID (NOD-*LtSz-scid/scid*) mice showed a significant proliferation of human cells but also the presence of fully mature circulating hRBCs (28%) with production of adult Hb [36]. Nevertheless, the percentage of circulating hRBCs decreases sharply until it becomes undetectable a few days after injection, being probably eliminated by macrophages of the recipient mouse [37]. Furthermore, to improved engraftment and differentiation of HSPCs in BM, researchers developed a humanized mice strains called NSGW41 [38]. Based on previous results [39,40], they designed a loss-of-function of KIT receptors into NOD/SCID *Il2rg*^−/−^ mice, which carry out ineffective erythropoiesis. This new tool provides higher and stable engraftment but also enhanced HSPCs differentiation and enucleation compared to NSG basic mice [38,41,42]. To avoid the phagocytosis of hRBCs by murine macrophages, they used clodronate-containing liposomes (CloLip) to eliminate them from the blood. However, despite a higher percentage of hRBCs in circulation, this method leads to incomplete differentiation [38]. Already described as a regulator of erythroblasts proliferation, differentiation, and enucleation, macrophages play an important role in erythropoiesis. Thus, it may be possible that their inhibition can explain the observed deficiency. These different approaches highlight the critical role of BM as well as the microenvironment during erythropoiesis.

## 3. Bone Marrow and Microenvironments

Viewed as the main site of hematopoiesis, BM is a complex tissue promoting the formation of specialized microenvironments or niches allowing the self-renewal of HSCs and their differentiation into myeloid or lymphoid fate [43]. Majority within the BM, HSCs, as well as the progenitors and precursors deriving from these cells, are accompanied by endothelial and neuronal cells but also by mesenchymal stem cells producing non-hematopoietic cell types also participating in the homeostasis of this area. These include osteoblasts, adipocytes, and mesenchymal stromal cells (MSC) [44,45]. Over the past 20 years, discovery of specific factors as well as the characterization of the cells present in BM has led to a better comprehension of the physiologic condition governing the hematopoietic niches but all that remains poorly understood.

### 3.1. HSCs Localization and Mesenchymal Stromal Cells

Long-term repopulating HSC are usually located around the vascular system, near to the sinusoids into the red BM, favoring their migration capacity to the circulation and the access of plasma components [46,47,48]. However, an important number of HSCs is also associated with arterioles, related to a quiescence status due to the presence of Schwann cells [49,50]. HSCs localization introduces the perivascular niches concept [46]. This concept was strongly supported by the identification of specific cells secreting SCF and the CXC-chemokine ligand 12 (CXCL12) that are crucial for HSC proliferation and maintenance. These soluble factors are locally secreted by LEPR^+^, NG2^+,^ and CXCL12-abundant reticular (CAR) stromal cells as well as by endothelial cells surrounding the vessels and, therefore, located near HSCs (Figure 1) [51,52,53,54]. The depletion of these factors in specific cells leads to HSCs depletion or reduction, showing their differential contribution between niches and their critical importance in stem cell proliferation and differentiation [52,55]. In addition, co-culture system was carried out to study the interaction between HSCs and the stromal layer, showing that cell contact promotes the cell cycle and expansion of HSCs [56]. Although the stromal cell function in HSC maintenance is now well-accepted, these studies did not show a specific role of these cells on the regulation of the erythroid differentiation. Nevertheless, to better define their role and optimize erythropoiesis in vitro, stromal cells have been used as feeder layer in co-culture protocols searching to recreate a physiological environment.

Kaufman et al. used hESCs (H1, H1.1, and H9.2 line) in co-culture with mouse stromal cell line S17 or mouse yolk-sac endothelial cell line C166 without exogenous growth factors or cytokines [57]. The approach is considered successful by the appearance of multiple hematopoietic lines after a few days of culture. Specifically, expression of the glycophorin A (GPA) and adult hemoglobin demonstrates the presence of terminal erythroid precursor. This study shows that stromal cells have a role in both the maintenance and differentiation of HSCs and represent a useful strategy for in vitro studies. In addition, another study has shown similar results using OP9 stromal cells and the addition of Flt3-L, interleukin 7 (IL-7), and IL-3 in the medium [58]. A few years later, a co-culture approach using mouse fetal liver-delivered stromal cells (mFLSCs) and cytokines addition successfully generated enucleated RBCs able to achieve hemoglobin switch and oxygen transport [59]. In addition, they raise the hypothesis that association with stromal cells is necessary for the maturation of progenitors. However, despite satisfying results, the hESCs use remains problematic from an ethical point of view.

For this reason, native hHSCs or iPSCs have been privileged in in vitro protocols. Using murine MS-5 stromal cell line or human mesenchymal cells, Giarratana’s team directed erythropoiesis of hHSCs derived from BM, CB, and PB with a three-steps protocol [18]. After an expansion and differentiation phase with the addition of SCF, IL-3, and EPO, cells are cultivated only with EPO on stromal layer for 3 days and without exogenous addition until the end. At the end of the assay, the results reveal a large scale of proliferation and differentiation with total enucleation, leading to fully mature and functional RBCs containing hemoglobin A. Nevertheless, depending on HSCs origin, rate and nature of hemoglobin vary. Whereas HSC derived from PB and BM contains more than 90% adult hemoglobin, those derived from cord blood carry mainly fetal hemoglobin (HbF) [18]. Based on these results, another team attempted to characterize the effect of MSCs in HSCs fate. Using MSCs isolated from patient’s bone marrow and HSCs from peripheral blood, they showed that, with or without cell contact, the stromal layer strongly influences the viability, proliferation, and fate of HSCs by promoting the myeloid lineage [60]. In addition, presence of IL-6 and especially G-SCF in the supernatant of stromal cell culture shown to be involved in the commitment to erythroid lineage [61]. Even if the enucleation percentage (40%) is less efficient than for the Giarratana’s studies, it remains more extensive than in most basic in vitro approaches, showing that MSCs may be involved in enucleation by direct contact or by molecule secretion. In the same years, a study compares the production of RBCs derived from hiPSC or hESCs using an OP9 coculture system. Although attempts to erythropoiesis with reprogrammed fibroblasts were inconclusive due to maturation and hemoglobin, they demonstrated that OP9 stromal cells could support the generation of RBCs derived from iPSCs at hPSC scale [62]. By the way, in most of the articles using HSCs or iPSCs, the stromal layer allowed the switch of hemoglobin, which was difficult to achieve with other in vitro approaches [18,31,62,63].

Mimicking the BM microenvironment with MSCs has become an approach used by many researchers and has shown to significantly improve in vitro results. However, BM is a complex set involving other important components.

### 3.2. Osteoblasts

Osteoblasts constitute an integral part of bone tissue and are undergoing extensive studies to characterize their function in the various processes governing the hematopoietic niche. Mainly derived from CAR/LepR^+^ cells, osteoblasts allow close contact with BM cells [64,65]. The role of osteoblasts is intrinsically linked to the formation of niches, as demonstrated by the suppression of the Osterix and VEGF factors involved in bone formation [66]. More recently, a strong balance was established between precursor and differentiated cells to regulate the space in the bone marrow and create niches [67]. In addition, technical improvements and new research approaches provide the opportunity to study more specifically the link between MSCs and osteoblasts in the microenvironment. In 2016, a three-dimensional co-culture cytokine-free was performed with BM MSCs, differentiated osteoblasts, and HSCs from CB [68]. These cells were seeded into bio-derived bone, creating a 3D system that mimics hematopoietic niches. This approach provides significant support on the quiescence, expansion, and differentiation potential of HSCs. Interestingly, they demonstrated the strong expression of SCF and CXCL12 but also the secretion of an extracellular matrix, implying a close relationship between all the components allowing the modulation of HSCs.

Although the role of osteoblast in the maintenance of all HSCs was unclear, they may provide a supportive environment for quiescent long-term repopulating cells at the endosteal surface. Described several years ago, osteoblasts can secrete various factors such as angiopoietin-1 (Ang-1) and thrombopoietin (THPO) (Figure 1), contributing to the quiescence of HSCs [69,70,71,72]. In addition, osteoblasts can affect differentiation. Conditional ablation of the osteoblast lineage in mice leads to an HSC-derived progenitor and precursor loss as well as HSCs decrease, showing an important role in hematopoiesis [73]. Moreover, osteoblast secret IL-7, a key signal for B lymphopoiesis but also the erythropoiesis when combined with EPO [74,75]. To study the mechanism by which osteoblasts dictate the destiny of HSCs, a co-culture was set up in 2013. Results have shown that osteoblasts support the later differentiation of HSPCs, but erythroid lineage is inhibited in favor of the lymphoid lineage [76]. Up to now, no studies have been carried out to study the potential supporting role of these cells during erythropoiesis. Further studies are necessary to carry out this process in vitro with a feeder layer, knowing that osteoblasts can secrete EPO, which is one of the most important factors for erythroid progenitors [75,77].

### 3.3. Extracellular Matrix

Although endothelial cells, MSCs, and osteoblasts are the main players in the bone marrow, extracellular matrix (ECM) is a huge part of this environment. Composed mainly of macromolecules like collagen, glycoproteins, and proteoglycans, this matrix surrounds different cells in the BM, giving the structure to the different niches, allowing the retention of multiple factors secreted principally by the stromal cells and supporting HSCs self-renewal [78,79,80]. The niche cells themselves produce the components of the ECM. During the maturation of erythropoietic cells, laminin has a supporting effect on erythroid cells by mechanically facilitating enucleation [60].

HSCs are, therefore, integrated into specialized niches surrounded by various cells and factors, which play an important role both in the maintenance of stem cells and in their destiny. However, within the BM, other niches leading to specific processes can be found, such as for erythroid cells. Extensively studied during the last few years, they provide a better understanding of the erythropoiesis process.

## 4. Erythroblastic Islands

The discovery of the environment that governs the progenitors and precursors of definitive erythropoiesis was achieved more than 60 years ago by Marcel Bessis [81]. He described a particular structure formed by a central macrophage surrounded by 5 to 10 differentiating erythroblasts and proposed that such organization, named as erythroblast island (EBI), would promote the proliferation and differentiation of erythroid lineage cells. In 1978, Mohandas and Prenant confirmed this discovery by three-dimensional reconstruction of the rat bone marrow [82]. Subsequently, extensive studies have been conducted to further understand and characterize this emerging niche.

### 4.1. Localization and Characterization

Although within the bone marrow the islands are evenly distributed, their composition differs according to the distance from the sinusoids [82,83]. CFU-E and early erythroblasts are located around the macrophages further away from the vessels, while later erythroblasts are closer to sinusoids, allowing the reticulocytes to be released into the circulation [83]. This supports the hypothesis of erythroblastic islands migration toward the sinusoids. Phenotypical characterization of central macrophages remains poorly defined and represents a challenge for researchers. Studies have been conducted to identify cell surface markers combination to distinguish them from other existing macrophages or monocytes. The most commonly used markers are F4/80 antigen, CD169, and vascular adhesion molecule-1 (VCAM-1) [84,85]. To increase precision of macrophages identification, other markers may be employed such as ER-HR3, LY6G, EPOR, CD11b [86,87]. However, the population of macrophages present in the BM appears to be heterogeneous, although the central macrophage seems to be phenotypically M2-like [87,88,89].

### 4.2. Function

Formation of erythroblastic island involves the binding between macrophages and CFU-E through cytoplasmic extensions and adhesive interaction. Specific interactions have been identified to play a key role in the formation and integrity of this organization: integrin-α4β1 binding/vascular adhesion molecule-1(VCAM-1), intercellular adhesion molecule-4 (ICAM-4)/integrin-αV, and erythroblast macrophage protein (EMP) or macrophage erythroblast attacher (MAEA) (Figure 1), present on both cells [90,91,92,93,94,95]. Moreover, EMP/MAEM binding could be involved in EBI migration due to the presumed involvement of EMP in the Erk1/Erk2 or PI3/Akt pathways as well as the regulation of gene expression associated with cell motility [96,97]. Furthermore, erythroblast/macrophage interaction appears to be a complex process that involves several more molecules than those described today. Recently, a study revealed a new partner, Ephrin/Eph receptor, that may be associated with the recognition between the cells and thus the formation of the EBI [98]. Reports dealing with the various interactions involved in this process highlight their significance in the primary function of the EBI, providing an optimal environment throughout the erythroblast proliferation and maturation stages [90,99,100]. This function is particularly crucial, enabling an increase in the number of cells generated during stress erythropoiesis [4,101]. The existence of positive and negative feedback between erythroblast/macrophage, erythroblast/erythroblast, and factors secretion allowing the balance between cell survival and apoptosis by the EBI are extensively discussed [102,103].

At the end of the terminal erythroid differentiation, Ortho-E enucleate produces the reticulocyte and the pyrenocyte. In this process, macrophages from EBI play a key role in favoring enucleation and phagocyting the pyrenocyte, whose elements will be recycled. During the orthochromatic stage, the cells undergo chromatin condensation and polarization of its nucleus [6,104,105]. Following this, microtubules and actin allow nuclear pyknosis and expulsion, which, respectively, forms a basket around the nucleus and leads to the formation of the contractile actin ring (CAR). It has been shown that macrophage, via the protein EMP, interacts with actin filaments and allows their normal distribution, as well as pyrenocyte binding for phagocytosis [3]. After enucleation, when the pyrenocyte is completely detached from the reticulocyte, phosphatidylserine exposure acts as an “eat me” signal [106]. Macrophages then engulf the pyrenocyte in a PtdSer-dependent manner through a MerTK-dependent mechanism and perform nucleus phagocytosis [94].

Additionally, a possible role in iron homeostasis was suggested following the discovery of ferritin exchange between erythroblasts and macrophages [107]. However, due to a lack of evidence, this hypothesis remains controversial [108].

### 4.3. Co-Culture

Over the years, macrophage/erythroblast co-culture began to emerge, but the main purpose was to find a suitable cell culture model to better understand their interaction. Interestingly, these methods may improve the performance of in vitro erythropoiesis, leading to the development of innovative approaches that combine optimization and understanding.

The first application of this approach was conducted in 1994 to investigate the molecules involved in the EBI interactions [90]. Using peripheral blood mononuclear cells and a two-phase culture, they succeeded in discovering a protein involved in the binding of EBI but also demonstrated that the association of erythroblasts/macrophages increased the proliferation and maturation of RBCs with 35% (±5%) enucleation [90]. This increase in enucleation compared to different in vitro cultures in absence of macrophages showed the benefit of this approach. Various articles subsequently demonstrate that co-culture can significantly increase the expansion of erythroblasts in a murine or human context [99,101,109,110]. All these studies followed different strategies to collect the macrophages. While Rhodes et al. reproduced EBI in vitro in a mouse anemia context with spleen cells including central macrophages [99], other studies used cells purified from human PBMCs. Interestingly, both CD34^−^ [101,109] or CD34^+^ [110] cells could be used, indicating that even though macrophages are phenotypically different, they still support erythropoiesis and contribute to the proliferation of erythroblastic progenitors. However, results shown by Hanspal and Hanspal regarding the increased differentiation of erythroblasts in the presence of macrophages is not reported in either of the articles mentioned rather demonstrating a delay in erythropoiesis [90]. Nevertheless, it is noteworthy that while most of studies were performed using basic medium with EPO and SCF, these authors used a 5637-cell line-conditioned medium (5637 CM). This medium was subsequently characterized as possessing significant levels of G-CSF, GM-CSF, and M-CSF [111]. It is now established that G-CSF could promote the differentiation of erythroblasts and GM-CSF as M-CSF promotes monocyte-macrophage differentiation, which could explain the differences in results between these articles. [61,112]. Beyond the selected method, this discrepancy leads to a debate about the significance of EBI during erythropoiesis in mammals but also about macrophage heterogeneity derived from PBMC.

As mentioned above, EBI-specific macrophages are not fully characterized and are exceedingly difficult to access for in vitro differentiation model. During monocyte-macrophage differentiation in vivo, there is a heterogeneous population of attached cells, which means that only a small portion could represent the central macrophage and thus reduce the final performance of erythropoiesis. Recent scientific advances have overcome the limitations thus through genetic programming technology to generate EI-like macrophages using iPSCs line and the safe harbor AAVS1 loci to integrate an KLF1 transgene [113]. Co-culture with CD34^+^ derived from cord blood shows that, with KLF1 induction, there is an increase in the production of erythroid cells and their maturation (10-fold). In addition, KLF1 transgene mimics a similar phenotype of EBI macrophage in vivo, by increasing their phagocytic activity as well as increasing the expression of specific factors affecting the maturation of erythroblasts, ANGPTL7, IL33, and SERPINB2. This strategy provides an interesting tool for the elucidation of terminal erythropoiesis as well as for large-scale production of mature RBCs for cell therapy but requires further studies on the functionality and hemoglobin.

## 5. Conclusions

As illustrated in Figure 1, the different cellular and molecular actors mentioned in this study play an important role in the regulation of HSC homeostasis and erythroblast differentiation. It shows that erythrocyte differentiation is controlled by multiple interactions in highly specialized environments such as the BM niche or the erythroblast island. These interactions lead to a complex physiological regulatory system based on the concentration gradient of cytokines and differentiation factors.

Today, despite progress in research, we are not able to draw clear conclusions on the existence of an appropriate cell culture model that combines all the criteria recreating a physiological environment. Nevertheless, important discoveries on bone marrow and microenvironments have been made in recent years, providing a better understanding of the physiological environment of erythropoiesis that may allow us to solve this problem. As a result, promising new tools have been developed, ranging from simple erythroid culture to 2D or 3D co-culture, allowing an increase in: the long-term expansion time of erythroid cells, the production of red blood cells, enucleated cells, and the presence of adult hemoglobin [18,20,32,42,68,113,114].

## Abbreviations

Ang-1	Antiopoietin-1
Baso-E	Basophilic erythroblast
BFU-E	Burst forming unit erythroid
BM	Bone marrow
BMSC	Bone marrow stromal cell
CAR	Contractile actin ring
CB	Coord blood
CFU-E	Colony forming unit erythroid
EBI	Erythroblastic island
ECM	Extracellular matrix
EMP	Erythroblast macrophage protein
EPO	Erythropoietin
ESC	Embryonic stem cell
GMP	Good manufacturing practice
GPA	Glycophorin A
HbF	Fetal hemoglobin
hESC	Human embryonic stem cell
HSC	Hematopoietic stem cell
iPSC	Inducible pluripotent stem cell
KLF1	Erythroid Kruppel-like factor 1
MAEA	Macrophage erythroblast attacher
MDS	Myelodysplastic Syndrome
mFLSC	Mouse fetal liver-delivered stromal cells
MSC	Mesenchymal stromal cell
Ortho-E	Orthochromatic erytroblast
PB	Peripheral blood
PBMC	Peripheral blood mononuclear cell
Poly-E	Polychromatic erythroblast
RBCs	Red blood cells
SCF	Stem cell factor
THPO	Thrombopoietin
